# DNIC-mediated analgesia produced by a supramaximal electrical or a high-dose formalin conditioning stimulus: roles of opioid and α2-adrenergic receptors

**DOI:** 10.1186/1423-0127-17-19

**Published:** 2010-03-19

**Authors:** Yeong-Ray Wen, Chia-Chuan Wang, Geng-Chang Yeh, Sheng-Feng Hsu, Yung-Jen Huang, Yen-Li Li, Wei-Zen Sun

**Affiliations:** 1 Graduate Institute of Clinical Medicine, College of Medicine, Taipei Medical University, Taipei, Taiwan; 2 School of Medicine, College of Medicine, Taipei Medical University, Taipei, Taiwan; 3 Department of Anesthesiology, Shin-Kong Wu Ho-Su Memorial Hospital, Taipei, Taiwan; 4 School of Medicine, Fu Jen Catholic University, Taipei County, Taiwan; 5 Graduate Institute of Acupuncture Science, China Medical University, Taichung, Taiwan; 6 Department of Anesthesiology, National Taiwan University Hospital, Taipei, Taiwan

## Abstract

**Background:**

Diffuse noxious inhibitory controls (DNIC) can be produced by different types of conditioning stimuli, but the analgesic properties and underlying mechanisms remain unclear. The aim of this study was to differentiate the induction of DNIC analgesia between noxious electrical and inflammatory conditioning stimuli.

**Methods:**

First, rats subjected to either a supramaximal electrical stimulation or an injection of high-dose formalin in the hind limb were identified to have pain responses with behavioral evidence and spinal Fos-immunoreactive profiles. Second, suppression of tail-flick latencies by the two noxious stimuli was assessed to confirm the presence of DNIC. Third, an opioid receptor antagonist (naloxone) and an α2-adrenoreceptor antagonist (yohimbine) were injected, intraperitoneally and intrathecally respectively, before conditioning noxious stimuli to test the involvement of descending inhibitory pathways in DNIC-mediated analgesia.

**Results:**

An intramuscular injection of 100 μl of 5% formalin produced noxious behaviors with cumulative pain scores similar to those of 50 μl of 2% formalin in the paw. Both electrical and chemical stimulation significantly increased Fos expression in the superficial dorsal horns, but possessed characteristic distribution patterns individually. Both conditioning stimuli prolonged the tail-flick latencies indicating a DNIC response. However, the electrical stimulation-induced DNIC was reversed by yohimbine, but not by naloxone; whereas noxious formalin-induced analgesia was both naloxone- and yohimbine-reversible.

**Conclusions:**

It is demonstrated that DNIC produced by different types of conditioning stimuli can be mediated by different descending inhibitory controls, indicating the organization within the central nervous circuit is complex and possibly exhibits particular clinical manifestations.

## Background

Nociception is dynamically regulated by endogenous modulation systems, and final pain perception depends on a balance between nociceptive stimulation and the processing networks. Diffuse noxious inhibitory controls (DNIC), among the networks, occur when a painful stimulus (i.e., a conditioning stimulus) in one body region suppresses another noxious response (i.e., a test stimulus) in a remote body region [1,2], and is an important mechanism to modulate the activations of nociceptive convergent neurons (wide-dynamic-range neurons) at spinal cord or trigeminal nucleus through an inhibitory pathway descending from the lower brainstem [1-4]. Nevertheless, interactions between conditioning stimuli and analgesic responses are largely in unclarity.

DNIC can be triggered by different types of conditioning stimuli, e.g., noxious heat, cold water [5-7], cold presser [8], a brief electrical stimulus [9], and a CO_2 _laser [10]. However, DNIC effects differ depending on the types of conditioning stimuli, and also on the quality, magnitude, and activated nerve fibers [1,2]. For instance, intense electrical stimulation sufficient to activate Aδ and C fibers or to induce pain showed heterotopic analgesia in animals [11-14] and humans [15,16]. In contrast, electroacupuncture (EA), another form of electrical stimulation using a much-lower intensity, also produce remote analgesic effects. It was therefore intriguing to investigate whether analgesic quality is differential between high-intensity (noxious) electrical stimulation and low-intensity EA-like stimulation.

Inflammation also results in pain; however, inflammatory pain-induced DNIC have seldom been studied. A formalin injection in the rat paw results in local inflammation and pain. It was reported that pinch-induced pain at the hindpaw was inhibited by formalin injection in the forepaw by evidence of decrease in Fos expression [17], a pain marker [18-20], suggesting a DNIC effect. However, the presence of DNIC in an inflammatory conditioning may be complicated because studies from monoarthritic animals [21] and rheumatoid arthritic humans [22] indicated that the arthritic duration (acute vs. chronic), pain pattern (evoked vs. constant), and applied pain type (mechanical or thermal) all caused different results in the second pain (test stimulation). Accordingly, the purpose of this study was to investigate underlying differences in DNIC responses to two conditioning stimuli, a supramaximal electrical stimulation and an injection of high-dose formalin, applied to the same area. Three sequential steps were undertaken to achieve this aim. First, identify nociceptive qualities of the two conditioning stimuli by behavioral observations and neural activations; second, compare heterotrophic analgesic effects (DNIC) between the two conditioning stimuli by the tail-flick test; and third, with a pharmacological approach, differentiate the recruited descending pathways involved in DNIC responses to the conditioning stimuli.

## Methods

### 1. Animal preparation and inhalational anesthetic technique

Male Sprague Dawley rats (250-350 g, BioLASCO Taiwan, Taipei, Taiwan) were housed in groups of two or three at 23 ± 1°C with a 12-h dark-light cycle with food and water available *ad libitum*. Studies were performed under approval of the Animal Care and Use Committee of Shin-Kong Wu Ho-Su Memorial Hospital, and strictly followed Guidelines for the Care and Use of Experimental Animals [23].

All experiments, except that in Methods section 2.2, were conducted under an previously reported anesthetic model [24]. In brief, rats were rapidly anesthetized in an acryl chamber containing halothane-soaked cotton, and then transferred to a transparent tube connected to a breathing circuit pre-filled with 0.75% halothane in pure oxygen with a flow of 2 L/min. The halothane concentration was monitored with a gas analyzer. A 10- to 15-min "induction period" was necessary to keep animals at a stable anesthetic level [24]. As long as stable tail-flick latencies (TFLs) were obtained, conditioning stimuli were begun, and anesthesia was maintained to the end of the study. Usually, animals recovered to a conscious, freely movable condition in 5 min after anesthesia removal.

### 2. Noxious conditioning stimulation

#### 2.1 The supramaximal electrical stimulation

The electrical stimulation was modified from our previous study [24]. One pair of stainless steel needles (30G) was inserted to a depth of 5 mm in the right acupoint Zusanli (ST36), a point located 5 mm inferolateral to the right fibular tuberosity and in the upper one-third of the anterior tibial muscle, and a reference point 10 mm below. Electricity was generated by a Grass S88 stimulator (Astro-med, Grass, Warwick, RI, USA) with two Grass constant current units to deliver the electric current of square pulses at 4 Hz with a 0.5-ms pulse width. The stimulating current was increased from a level producing local muscle twitching at about 0.3-0.4 mA (twitch intensity, or abbreviated as TI) to the target intensities within 5 min. Three target intensities were 10×TI (3-4 mA; and named as E10), 20×TI (6-8 mA; E20) and a supramaximal intensity (E50), which was as high as the animals could tolerate (usually 50-80×TI or > 20 mA). Total stimulating period was 30 min. Characteristic rhythmic dorsiflexion of the stimulated hind limb was always seen.

#### 2.2 Intramuscular (i.m.) formalin injection and weighted pain scores

To determine which concentration of i.m. formalin would cause a hyperalgesic effect analog to that of an intraplantar (i.pl.) injection, weighted pain scores were used to measure the responsiveness of graded concentrations from 5% to 20%. First, we tested the appropriate concentration, volume, and depth of formalin required to induce nociceptive behaviors. In conscious rats, 100 μl of 5%, 10%, or 20% formalin, or normal saline (abbreviated as Fm5, Fm10, Fm20, and NS, respectively) was injected into the right Zusanli point. An injection with 50 μl of 2% formalin (Fp) in the plantar surface of the right hindpaw served as a positive control group. After injection, the rats were transferred to an open iron-wire cage for an 1-h evaluation using a modified weighted pain score method [25]. The scores of an early phase (0-15 min), late phase (20-60 min), and total phase (0-60) were separately calculated for comparison.

To confirm the spread of the injection, 20 μl of methylene blue was added to the formalin solution in some rats, and the extent by which the injectate spread in muscles was examined after the animals were sacrificed.

#### 2.3. Neuronal activations by conditioning stimuli: Fos immunohistochemistry

To investigate neuronal activation by the conditioning stimuli, Fos-immunoreactive (Fos-ir) profiles in the lumbar dorsal horns were analyzed. Rats from the Fm20, E20, and E50 groups were sacrificed at 90 min after the beginning of the conditioning stimulation. Rats were intraperitoneally injected with an overdose of 650 mg/kg chloral hydrate (Kanto Chemical, Tokyo, Japan) and transcardially perfused with 250 ml of saline followed by 350 ml of 4% paraformaldehyde in 0.1 M phosphate-buffered saline (PBS, pH 7.4, 4°C). The L2-L5 spinal segments were removed, post-fixed in the same paraformaldehyde solution at 4°C for 6 h, and cryoprotected in 30% sucrose at 4°C for 48-72 h. Frozen sections were cut in a cryostat (30 μm) and collected in PBS as free-floating sections. They were then incubated with primary rabbit polyclonal anti-Fos antiserum (1: 1500, Santa Cruz Biotechnology, Santa Cruz, CA, USA), and diluted in 0.1 M PBS containing 3% normal goat serum and 0.3% Triton X-100 at 4°C for 24 h. After washing in PBS, sections were incubated with a biotinylated goat anti-rabbit secondary antibody (1: 200, Vector Laboratories, Burlingame, CA, USA) in PBS for 1 h at room temperature, and subsequently reacted with the avidin-biotin-peroxidase complex (Elite ABC kit, Vectastain^®^, Vector Laboratories) for 1 h at room temperature. After rinsing in 0.1 M PBS for 20 min, sections were reacted with a 3,3'-diaminobenzidine tetrahydrochloride solution in PBS containing hydrogen peroxide and nickel (Peroxidase substrate kit, Vector Laboratories) for 6 min. All sections were mounted on gelatin-dubbed slides, air dried, and protected with a coverslip for inspection under a light microscope.

Sections were examined under a Nikon E600 microscope (Tokyo, Japan) using a dark field to determine the segmental levels [26] and a light field for cell counting. The spinal dorsal horn was divided into three regions: (1) the superficial layer (laminae I/II); (2) the nucleus proprius (laminae III/IV); and (3) the deep layer (lamina V). Immunoreactive neurons, which had deep staining distinguishable nuclei from the background, were counted by laminae. For each animal, at least 8-10 sections of each segment were examined, counted, and averaged by segment. Antibody specificity and immunostaining were tested by omission of the primary antibodies. The evaluator who did the counting was blind to the group allocation of the samples.

### 3. DNIC effect

#### 3.1. Tail flick test as a test stimulus to evaluate analgesic effect

The DNIC effects were analyzed by tail flick test. With strict control of the ambient temperature at 23°C, the rat tail was heated at the distal one-third by radiant light from a focused projection bulb in an algesic device (MK-330B, Muromachi Kikai Co. Ltd., Tokyo, Japan). The baseline "tail flick latency" (TFL) was 8-10 s in naive rats, and the tail was passively removed at 20 s, as the "cutoff limit". The "basal latency" was measured after the anesthetic induction period and before the conditioning stimulus, or the time point 0. The "test latency" at each time point was an average of two successive tests, separated by 2 min, without pause of electrical stimulation. The maximal possible effect (MPE) was calculated as: MPE% = [(test latency - basal latency)/(20 - basal latency)] × 100%.

#### 3.2. DNIC effects produced by two conditioning stimuli

**Figure 1:**
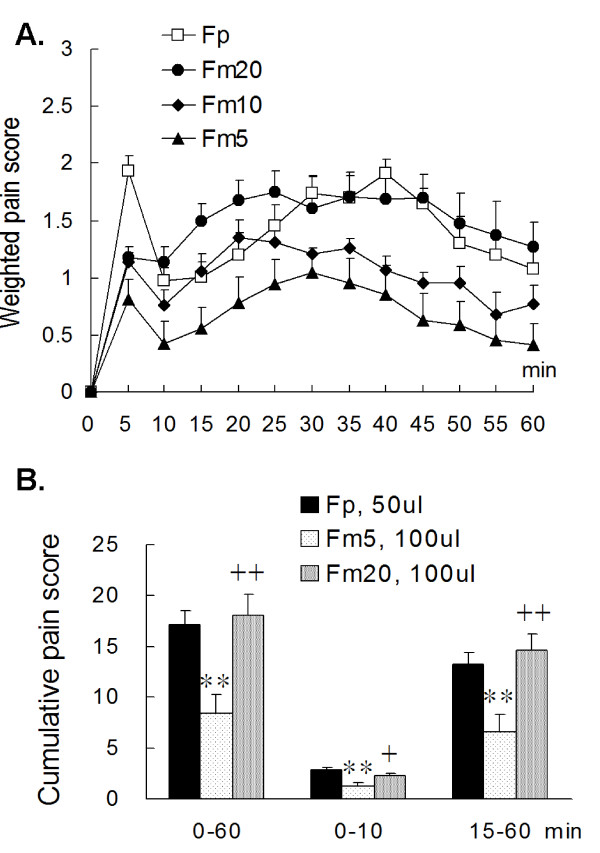
**Weighted pain score **[24,25]**of a formalin injection in the hind limb**. (A) An i.m. injection of 100 μl of 5% (Fm5), 10% (Fm10), or 20% (Fm20) formalin in the anterior tibial muscle induced dose-dependent pain scores and a biphasic pain pattern similar to that of a subcutaneous plantar injection (2%, 50 μl, Fp). The Fm20 group had a lower pain score in the early phase and fewer flinch responses for the entire period compared to the Fp group. However, the Fp and Fm20 groups had similar highest pain scores in the late phase. (B) The Fm20 group showed no statistical difference in the cumulative pain score from that of the Fp group, indicating the muscular injection with 100 μl of 20% formalin resulted in a strong noxious reaction. Rat numbers: Fp = 9, Fm5 = 7, Fm10 = 6, Fm20 = 7. ** *p *< 0.01 vs. Fp; ^+ ^*p *< 0.05, ^++ ^*p *< 0.01 vs. Fm5; one-way ANOVA with Bonferroni's *post hoc *test.

Five groups were included: (1) a control group (C) in which rats were inserted with needles but received no electrical stimulation; (2) an E10 group in which rats were given electrical stimulation at 10×TI; (3) an E20 group in which rats were given electrical stimulation at 20×TI; (4) an E50 group in which rats were given supramaximal electrical stimulation at 50-80×TI, or as high as the rats could tolerate; and (5) a Fm20 group in which rats were given an i.m. injection of 100 μl of 20% formalin in the right ST36 acupoint. All experiments were conducted under the same conditions of equal anesthetic levels and periods, basal latencies, and TLF time points (Fig. 1). In particular, our anesthetic device allowed three rats to be simultaneously anesthetized, so three groups (electrical, formalin, and control) could be matched under identical conditions and environmental biases would be greatly decreased. Each group contained at least nine rats. Rats were sacrificed at the end of the study for immunostaining.

### 4. The mechanistic study of DNIC-mediated analgesia

To differentiate mechanisms underlying DNIC between the electrical and formalin stimulations, involvement of inhibitory pathways were examined by neurotransmitter antagonists. Two agents were used: naloxone (Genovate Biotech, Hsinchu, Taiwan), an opioid receptor antagonist, and yohimbine (Sigma-Aldrich, St. Louis, MO, USA), a selective α2-adrenoreceptor antagonist. Naloxone was intraperitoneally injected twice, 2 mg/kg at time point -15 and 1 mg/kg at time point 30. Yohimbine was intrathecally (i.t.) injected with a dose of 30 μg in 20 μl of saline at the time point -15. The i.t. injection is a single bolus technique at the L5-L6 interspace using a 26-gauge needle and microsyringe (Hamilton, Reno, NV, USA) described elsewhere [27]. Both conditioning stimulations were tested with one of the antagonists and were compared with data of the control groups injected with an equal volume of saline vehicle. At least seven rats were included in each group.

### 5. Data analysis

All quantitative data are expressed as the mean ± standard error of the mean (SEM). Cumulative values of weighted pain scores at 0-15, 20-60, and 0-60 min, as well as cumulative TFLs over 0-90 min were transformations of the area under curve (AUC). The averaged TFL at each time point, AUCs, and Fos-ir cells were compared with one-way analysis of variance (ANOVA) followed by *post hoc *Bonferroni's test or Student's *t*-test. A value of *p *< 0.05 was considered statistically significant.

## Results

### 1. Supramaximal electrical stimulation and i.m. formalin injection induced noxious behaviors

#### 1.1. Supramaximal electrical stimulation was noxious

It is apparent that noxious behaviors were shown in the halothane-anesthetized rats subjected to the supramaximal electrical stimulation. When the electrical intensity exceeded 50×TI, a profile of characteristic behaviors including vigorous leg withdrawal, shaking off of the stimulating needles, and/or turn of body in the tube were observed. This finding was consistent with our previous study that most conscious rats cannot tolerate electrical intensities beyond 10×TI and exhibit similar behaviors [24]. Though the rats in the current study were anesthetized, it was believed that the E50 stimulation were still noxious enough to induce neuronal reactions.

#### 1.2. An adequate concentration of injected formalin to induce pain

Formalin injected into the plantar surface induced stronger pain than injection into the muscles. Biphasic pain pattern, typically seen after i.pl. formalin, was also observed after i.m. injection (Fig. 1), however, some differences in behaviors were shown. Intramuscular formalin induced much fewer flinch activities (pain score = 3) but longer hind paw elevation (pain score = 1-2) compared to those after i.pl. injections.

Dose-dependent hyperalgesic responses were shown in the i.m. formalin groups from concentrations of 5% to 20%. In the Fm20 group, pain in the early phase was not so evident, but pain score in the late phase was high because of persistent hind paw elevation. Data analysis showed that the Fm20 and the Fp groups had comparable maximal pain (1.90 ± 0.25 at 35 min in Fm20 *vs*. 1.91 ± 0.11 at 40 min in Fp, Fig. 1A) and comparable cumulative scores in the late phase (14.56 ± 1.71 for the Fm20 vs. 13.23 ± 1.12 for the Fp, *p *= 1.00) (Fig 1B). In the early phase, the Fm20 group had a lower score than the Fp group but the difference was insignificant (*p *= 0.50). Therefore, an i.m. injection of 100 μl of 20% formalin was proved to be noxious and this dose was chosen as a conditioning stimulus.

The spread of methylene blue was examined in four rats. Dense deep-blue staining was confined to the deep layer of the anterior tibial muscle and was scarcely dispersed through the interosseous membrane. No blue staining was found in the posterior calf muscles.

### 2. Supramaximal electrical stimulation and i.m. formalin both induced a significant increase in Fos expression in the spinal dorsal horns

**Figure 2:**
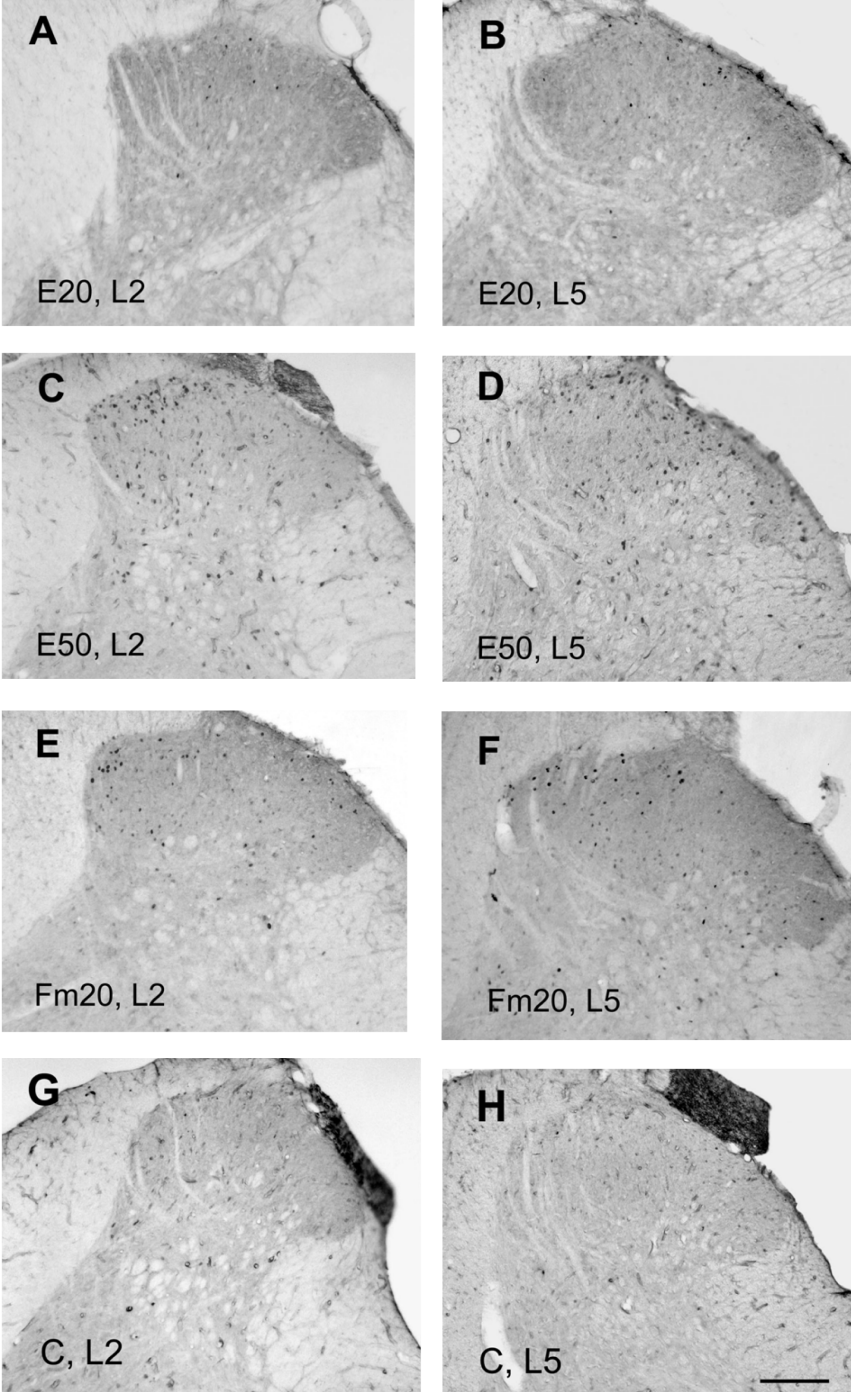
**Rostrocaudal distribution of Fos-immunoreactive (Fos-ir) neurons after two conditioning noxious stimulations**. Fos-ir neurons were identified at the L2 (A, C, E, G) and L5 (B, D, F, H) spinal dorsal horn at 90 min after the E20 and Fm20 stimulations. The groups shown in the figure are: the E20 (A, B), E50 (C, D), Fm20 (E, F), and control groups (G, H). Fos-ir neurons were few in the control and E20 rats at all segments (A, B, G, H). Formalin (Fm20) and supramaximal electrical (E50) stimulation induced Fos expression in L2 and L5 superficial laminae (C-F). In comparison, E50-induced Fos-ir neurons were densely distributed at the medial one-third of the L2 superficial dorsal horn (C), whereas Fm20-induced expression was relatively loosely distributed in the superficial dorsal horns (E). Scale bar = 100 μm.

**Figure 3:**
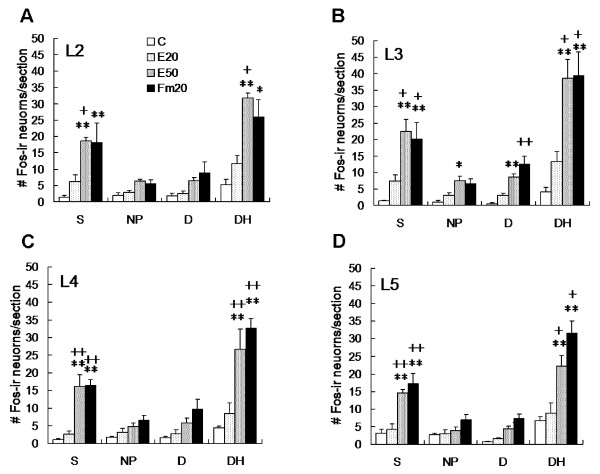
**Numerical analysis of Fos-ir neurons at the side ipsilateral to the conditioning stimulus**. Fos-labeled neurons were significantly higher in the Fm20 and E50 groups than in the control and E20 groups regardless of the spinal segment. No significant difference was found between the C and E20 groups, or between the Fm20 and E50 groups for all segments and laminae. Topographically, E50-induced Fos expression was higher in the higher segments (L2 and L3) than in the lower lumbar segments (L4 and L5). S, superficial laminae I/II; NP, nucleus proprius, laminae III/IV; D, deep laminae V; DH, dorsal horn. Rat numbers: C = 6, E20 = 6, E50 = 5, Fm20 = 6. * *p *< 0.05, ** *p *< 0.01 vs. C; ^+ ^*p *< 0.05, ^++ ^*p *< 0.01 vs. E20; one-way ANOVA with Bonferroni's *post hoc *test.

Whether the two conditioning stimuli produced different neuronal responses were examined with a profile of Fos-ir expression in the spinal dorsal horn. As shown in Fig. 2, rats in the control and E20 groups (low-intense stimulation) exhibited very few Fos-ir profiles from the L2 to L5 dorsal horns (Fig. 2A, B, G, H); however, the E50 and Fm20 groups showed marked Fos expressions in the superficial laminae (Fig. 2C, E), which were significantly higher than those of the control (*p *< 0.01) and E20 groups (*p *< 0.05 or 0.01, Fig. 3) at L2-L5 segments (Fig. 3). In comparison, Fos expression of the E50 group was densely distributed in the medial half of the superficial laminae of the L2-L3 segments, whereas Fos in the Fm20 group was loosely scattered in the superficial laminae of the same segments. In the lower L4-5 segments, Fos-ir cells of both the E50 and Fm20 groups were evenly expressed in superficial layers (Fig. 2D, F). The activated patterns of postsynaptic neurons were thus shown differently between the two conditioning stimulations.

### 3. Both noxious stimulations produced DNIC

#### 3.1. Electrical stimulation prolonged TFLs in an intensity-dependent manner

**Figure 4:**
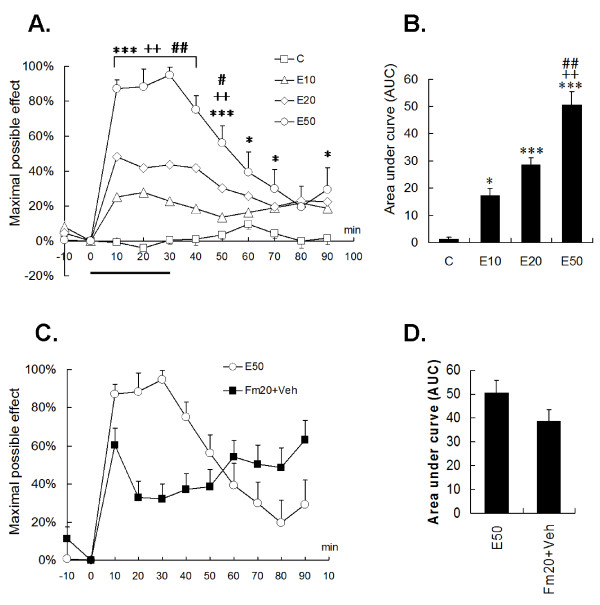
**Effect of noxious electrical and formalin stimulation-induced DNIC on the tail-flick latency**. (A, B) Halothane-anaesthetized rats were grouped into sham needles (C), E10 (10× twitch intensity), E20, and E50 (> 50×) electrical stimulation of the right ST36 acupoint. Changes in tail-flick latencies were compared by the "maximal possible effect". Control rats exhibited consistent tail-flick latencies without anesthetic influence. The electrical stimuli produced intensity-dependent analgesia on the tail reflex and showed maximal DNIC effects within the stimulation period. Notably, E50 elicited a strong and long analgesic effect (B). (C, D) A high-dose formalin injection (Fm20) caused a distinct DNIC pattern from E50 stimulation in tail-flick suppression. However, total pain summation (area under the curve) indicated no statistical difference between E50 and Fm20 (D). The horizontal thick bar indicates the electrical stimulating period. Rat numbers: C = 11, E10 = 10, E20 = 11, E50 = 9, Fm20 = 10. * *p *< 0.05, *** *p *< 0.001 vs. C; ^++ ^*p *< 0.01, ^+++ ^*p *< 0.001 vs. E10; ^# ^*p *< 0.05, ^### ^*p *< 0.001 vs. E20; one-way ANOVA with Bonferroni's *post hoc *test.

Under constant anesthesia, the control group maintained stable TFLs for 90 min, and graded electrical stimulation produced intensity-dependent suppression on tail-flick withdrawals during and after the stimulation period (Fig. 4A, B). E10 stimulation, using an EA-like intensity, mildly prolonged the TFL from time 0 to 40, and showed an after-effect during time 80 to 90. The maximal MPE of the E10 at time 20 was significantly higher than that of the control (*p *< 0.001). The supramaximal E50 stimulation produced strong analgesia for over 90% withdrawal inhibition at time 20-30, followed by a prolonged after-effect. Nevertheless, the E50 rat did not show traumatic signs, such as licking, elevating, limping, or local tissue inflammation at 1 wk of follow-up.

#### 3.2. Formalin injection produced a different pattern of tail flick depression

Noxious formalin (20%, 100 μl) suppressed tail withdrawal with a pattern differed from that of E50 stimulation. Immediate and short-lived analgesia occurred in the first 10 min after the injection, followed by increasing suppression of the tail-flick response (Fig. 4C). Obviously, the DNIC effect was correlated with an inflammatory process of the tibial muscle. This formalin-induced ongoing pain differed from the E50-evoked short-term pain, because the latter depends on the existence of electrical stimulation. Even though the current data revealed that both stimuli may have distinct DNIC patterns, there were no differences in the cumulative pain scores (Fig. 4D).

### 4. DNIC induced by supramaximal electrical and noxious formalin stimulation were mediated by different inhibitory pathways

**Figure 5:**
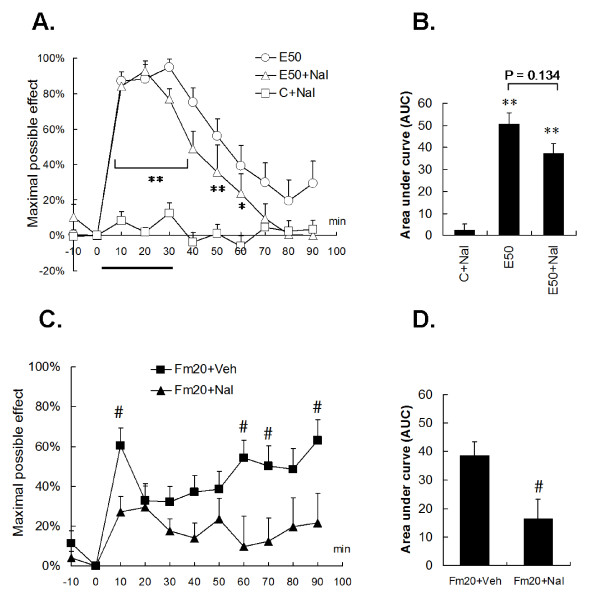
**Contribution of the opioidergic pathway to DNIC**. The opioid receptor was antagonized by a 2 mg/kg intraperitoneal naloxone injection at time point 15 and 1 mg/kg at time point 30. Naloxone itself did not alter the tail-flick latency in the control group (A, the C+Nal line). (A, B) The E50-induced DNIC was not reversed by naloxone administration, whereas the Fm20-induced DNIC was reversed in both the early and late phases by time point-to-point comparisons (C) or by total pain summation (D). Veh, saline; Nal, naloxone. The horizontal thick bar indicates the electrical stimulating period. Rat numbers: E50+Veh = 9, E50+NAL = 7, C+NAL = 9, Fm20+Veh = 10, Fm20+Nal = 9. ** *p *< 0.01, *** *p *< 0.001 vs. C+Nal; ^# ^*p *< 0.05 vs. Fm20+Nal; one-way ANOVA with Bonferroni's *post hoc *test.

Interestingly, the naloxone injection did not significantly reverse E50-induced DNIC in tail-flick responses. No statistical difference was found between the E50 and E50+Nal groups, regardless of the time-to-time comparison or cumulative analgesic calculation (E50 vs. E50+Nal, *p *> 0.05) (Fig. 5A, B). Meanwhile, it was shown that naloxone *per se *did not affect basal TFLs in the control. On the other hand, naloxone evidently reversed Fm20-induced DNIC, and antagonism was shown in the early (time 10) and late (time points 60, 70, and 90) periods (all *p *< 0.05, Fig. 5C). The Fm20+Nal group had a significantly lower area under the curve (AUC) than the Fm20 group by 57% (*p *< 0.05) (Fig. 5D). The results showed that noxious formalin, but not E50 stimulation, produced an opioid-dependent DNIC.

**Figure 6:**
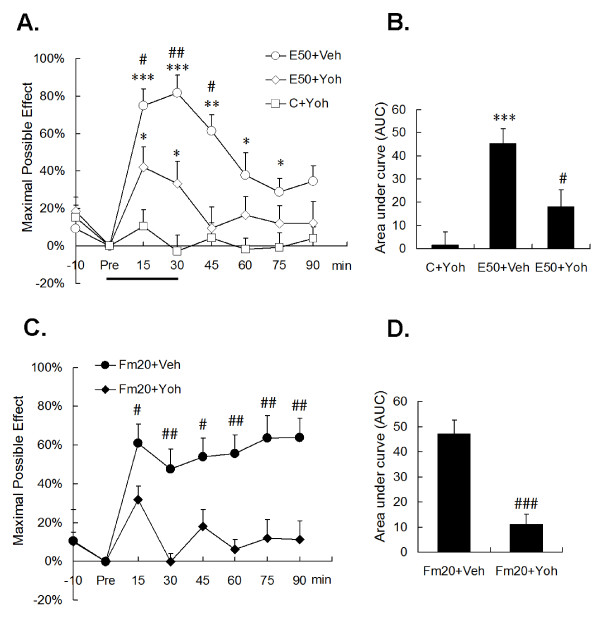
**Contribution of the α2-adrenergic pathway to DNIC**. The α2-adrenergic receptor was antagonized by an intrathecal yohimbine injection of 30 μg in 20 μl of saline, 15 min before the conditioning stimulus. Yohimbine did not alter the tail-flick latency in the control group (A, C+Yoh line). Unlike naloxone, yohimbine had the ability to reverse the DNIC effect produced by both E50 (A, B) and Fm20 (C, D). Veh, saline; Yoh, yohimbine. Rat numbers: E50+Veh = 9, E50+Yoh = 10, C+Yoh = 10, Fm20+Veh = 9, Fm20+Yoh = 9. * *p *< 0.05, ** *p *< 0.01, *** *p *< 0.001 vs. C+Yoh; ^# ^*p *< 0.05, ^## ^*p *< 0.01 vs. E50+Yoh (A, B) or vs. Fm20+Veh (C, D), respectively; one-way ANOVA with Bonferroni's *post hoc *test.

In contrast, the selective α2 receptor antagonist, yohimbine, showed a different action. Intrathecal yohimbine did not affect the basal TFLs in the control; however, DNIC of both conditioning nociception were significantly attenuated by i.t. administration of 30 μg yohimbine (Fig. 6). In both the E50 and Fm20 groups, yohimbine reversed DNIC for long-lasting periods (Fig. 6A, C). The analgesic summation (AUC) demonstrated a strong DNIC reversion of over 60% in both groups (In E50, from 45.35 ± 6.27 to 17.99 ± 7.28, *p *< 0.05; in Fm20, from 47.06 ± 4.15 to 10.99 ± 4.06, *p *< 0.001). The results proved that the α2-adrenergic pathway is involved in DNIC produced by either noxious electrical or formalin stimulation.

## Discussion

Our study on DNIC from two different types of conditioning stimuli, the supramaximal electrical stimulation and high-concentration formalin injection, reveals several findings. First, under a minimal-stress anesthetic condition, the supramaximal electrical stimulation, usually > 20 mA, induced much-stronger suppression of the tail withdrawal reflex than a low-intensity EA-like stimulation; second, formalin injection-induced muscular pain also elicited DNIC; third, noxious electrical and formalin stimulation induced Fos expression with a distinct topographical distribution in the spinal dorsal horns; and fourth, most importantly, the two conditioning stimuli triggered distinct underlying inhibitory pathways.

### The DNIC behaviors differed between the two conditioning stimuli

DNIC has been suggested to be dependent on the conditioning stimuli of different qualities, noxious intensities, durations, and locations [9,28-31]. We found an intensity-dependent inhibition of the tail flick reflex by graded conditioning electrical stimulations in this and a previous study [24]. In addition, we also showed different DNIC responses to various conditioning stimuli. The maximal DNIC effect in the E50 group appeared at the end of the electrical stimulus, whereas in the formalin group, the effect exhibited two peaks, one at the beginning and the other at the end of observation. The results apparently reflect a correlation between DNIC and noxious levels of the conditioning stimuli.

The activated peripheral nociceptors and projecting neurons by electricity and formalin may differ, which can partially explain the variations in DNIC. It is possible that E50 excites certain groups of mechano-receptors concentric to the electrical field, while injected formalin might diffuse to a broader region in the muscles and sensitize different groups of mechano- and chemo-receptors. In the meanwhile, the variation in the spinal Fos distribution provides additional evidence that post-synaptic neurons were differentially activated by both stimuli. Although Fos-expressing mapping is insufficient to justify the nociceptive quality, our immunostaining data are still informative at disclosing variations in the activation of CNS pathways.

### Supramaximal electrical stimulation-induced DNIC is naloxone irreversible, but yohimbine reversible

The supramaximal electrical stimulation in this study is an extrapolated example of EA, an intentional design which can be compared to our previous study [24]. The endogenous opioid system is a pivotal mechanism in EA analgesia [32-34] and also contributes to DNIC [35,36]. However, whether DNIC is involved in EA analgesia is quite controversial [16,31,37,38]. Moreover, it was suggested that different mechanisms could be triggered to suppress evoked potentials and tooth pain when the intensities increased from just activating large afferent A fibers to sufficiently recruiting C fibers [9,29].

In contrast to the concept that naloxone inhibited EA analgesia [24,39,40], we did not find a naloxone-reversible DNIC in the supramaximal E50 group. Since the supramaximal electrical stimulus activated broader neural circuits (all types of sensory afferents) and brain areas than did the lower-intense stimulations like EA (which only activated Aβ and Aδ fibers), it is presumed that as the electrical intensity increases from low (maybe no pain or only minimal pain) to high (strong pain), there is a shift in the triggered mechanisms in the central nervous system. Despite there were arguments whether endogenous opioids participating in acupuncture in rats [41-44], rabbits [45], and humans [46,47], our data in the first instance suggest that it is better to have a clear demarcation of the electrical intensity by which the underlying mechanisms of DNIC analgesia and acupuncture analgesia may differ.

**Figure 7:**
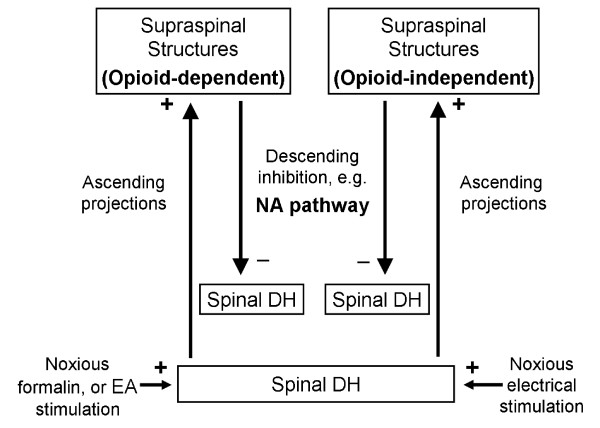
**A scheme of the proposed DNIC circuitry activated by noxious electrical or formalin stimulations**. Both noxious stimuli at the hindlimb can excite projection neurons in the corresponding spinal dorsal horn (DH) to activate descending inhibitory systems (e.g. noradrenergic pathway, NA) in the supraspinal structures to inhibit the noxious excitability in different spinal segments, e.g. the tail. In comparison, noxious formalin and electroacupuncture (EA), which is a low-intense electrical stimulation and may not be DNIC-mediated, produces analgesia through NA- and opioid-dependent actions (left upper panel), whereas the analgesic effect of noxious electrical stimulus may not depend on activation of opioid receptors (opioid-independent, right upper panel). Symbols +: excitation; -: inhibition.

On the other hand, yohimbine could reverse, though partially, E50-induced DNIC analgesia. In another study, when rats received 10× EA, the analgesia on an ankle sprain was reversed by yohimbine and phentolamine, a non-selective α antagonist, but not by terazosin, an α1 adrenergic antagonist [48]. Therefore, it was shown that descending analgesia of electrical stimulation is comprised, at least partly, of α2-adrenoceptor-mediated inhibition regardless of the stimulating intensity at a noxious or innocuous level. Taken together, electricity-triggered analgesia is classified into at least two mechanisms: an opioid-related mechanism predominating at low intensities and a α2 adrenergic system covering a much-wider range of intensities (Fig. 7).

### Noxious formalin-induced DNIC is both naloxone and yohimbine reversible

We found that high-dose formalin induces local pain and triggers DNIC which is reversed by naloxone. The formalin injection caused local inflammation and tissue injuries including muscles, fascia, vessels, and/or nerves. In addition to direct sensitization of the central endogenous opioid system, the inflammation activated release of proinflammatory cytokines and chemokines, and enhanced the production of leukocyte-derived opioid peptides [49,50]. For instance, Freund's adjuvant-induced inflammation showed an peripheral action of leukocyte-derived β-endorphin, met-enkephalin, and dynorphin on the μ, δ, and κ receptors, respectively, and opioid-mediated antinociception [51]. Therefore, the naloxone-reversible component in formalin-induced DNIC could include both central and peripheral opioid actions because intraperitoneal injection of naloxone could be systemically absorbed. By no means, it is demonstrated that formalin-induced DNIC consists of opioid and non-opioid mechanisms, and the latter may be inflammation-independent as in noxious electricity-induced DNIC and α2-recptor mediated (Fig. 7).

Descending inhibitory modulation mediated through spinal α2 receptor activation has been largely reported. Electrical stimulating peripheral Aδ and C fibers [52,53] or central noradrenergic cells [54] were found to trigger the descending adrenergic system and release norepinephrine in the spinal cord. Accumulating studies demonstrated that spinal norepinephrine administration [55-58] induced powerful antinociception or inhibited the amplitude of monosynaptically evoked A delta-fiber and C-fiber excitatory postsynaptic currents in a whole cell patch clamp technique [59]. However, spinal and pontine α2-adrenoceptors have opposite effects on pain-related behavior in neuropathic animals [60]. The current study provides additional evidence that a formalin injection stimulates yohimbine-reversible heterotopic analgesia. Conclusively, it is suggested that activation of the descending α2-adrenergic pathway may be a universal mechanism in DNIC effect through a variety of conditioning stimuli [56,61,62].

### Formalin-induced noxious inhibition or facilitation

Central nociceptive activation following peripheral inflammation should be meticulously interpreted based on various conditions. Contradictory results may be obtained because the opposing forces of descending modulations (facilitation *vs*. inhibition) can be simultaneously activated [3,63]. This study revealed that high-dose formalin induced DNIC analgesia. However, nociceptive hypersensitivity with a receptive field expansion is another probable consequence of prolonged inflammation due to long-term potentiation [64]. In rodents with acute monoarthritis (< 48 h), inhibition of trigeminal convergent neurons was produced by mechanical or thermal stimulation of the arthritic joint, whereas chronic arthritis of over 3 wk did not show a DNIC effect [21]. A human study revealed no difference in DNIC responses among patients with rheumatoid arthritis of over 5 years, < 1 year, and healthy controls. However, those 5-yr arthritic patients showed more pressure allodynia on the non-painful thighs than did 1-yr patients, indicating higher sensitization, but not inhibition, of somatosensory functions [22]. In these studies, pain duration played a critical role in determining the balance skewed to either side of two opposing forces. Unfortunately, many other factors, such as noxious qualities (e.g., inflammatory *vs*. neuropathic), can bias this tug-of-war. Therefore, more studies are necessary before introducing DNIC in clinical treatment.

### Clinical implications

The current DNIC study throws some light on EA application. For a long time, the acupuncture "dose" was efficacy relevant but remains a vague idea. Because acupuncture stimulating strength is largely empirical and the analgesic effect is also affected by psychological stressors such as nervousness, anxiety, and fear [34,65-68], it is difficult to predict the effectiveness of acupuncture in awake humans.

We found that stronger EA stimulation was more effective. Since EA analgesia is weak, equivalent to a morphine dose of 0.5 [69] to 2 mg/kg [24], increasing the electrical intensity can possibly produce a higher effect. For this reason, patients under general anesthesia are a suitable group of subjects to apply strong EA stimulation. This assumption agrees with our clinical study that preoperative high-frequency EA, which accumulated greater energy output, resulted in less morphine consumption than did the lower-frequency EA in the postoperative period [70]. Certainly, the highest limit of electrical power without causing tissue damage should be determined beforehand.

## Conclusions

DNIC is a well-known physiological phenomenon; however, its clinical value and application are unclear. This animal study provides information of differential consequences and mechanisms produced by two qualities of conditioning stimuli, supramaximal electrical stimulation and a noxious formalin injection. Clinical implications of the two noxious stimuli are respectively discussed on the basis of inflammatory states and perioperative analgesia. We suggest that greater understanding of DNIC analgesia, by structuring the complex descending circuitry with specific mechanisms under different conditioning, will help turn this theory into a useful clinical pain control application.

## Competing interests

The authors declare that they have no competing interests.

## Authors' contributions

YRW and WZS conceived of the study, designed and performed the experiments, analyzed the data, and wrote the manuscript. CCW participated in analyzing and revised the manuscript. GCY and SFH helped to design and coordinate the study, and participated in drafting the manuscript. YLL and YJH carried out the behavioral observations of the experiments. All authors read and approved the final manuscript.
